# Injection of YiQiFuMai powder protects against heart failure via inhibiting p38 and ERK1/2 MAPKs activation

**DOI:** 10.1080/13880209.2022.2038207

**Published:** 2022-03-04

**Authors:** Yongwei Nie, Yanxin Zhang, Zhi Li, Meixu Wan, Dekun Li

**Affiliations:** a School of Medicine, Nankai University, Tianjin, China; b Tianjin Tasly Pride Pharmaceutical Co., Ltd., Tianjin, China; c Tianjin Key Laboratory of Safety Evaluation Enterprise of TCM Injections, Tianjin, China

**Keywords:** Traditional Chinese Medicine, kinase inhibition, cardiac fibrosis

## Abstract

**Context:**

Injection of YiQiFuMai (YQFM) powder, a modern Chinese plant-derived medical preparation, has a therapeutic effect in heart failure (HF). However, its therapeutic mechanism remains largely unknown.

**Objective:**

To investigate the molecular mechanisms of YQFM in HF.

**Materials and methods:**

Kinase inhibition profiling assays with 2 mg/mL YQFM were performed against a series of 408 kinases. In addition, the effects of kinase inhibition were validated in cardiomyocyte cell line H9c2. *In vivo*, HF with reduced ejection fraction (HFrEF) was induced by permanent left anterior descending (LAD) coronary artery ligation for 6 weeks in male Sprague-Dawley rats. Then, HFrEF mice were treated with 0.46 g/kg YQFM or placebo once a day for 2 weeks. Echocardiography, immunohistochemistry, histological staining and Western blotting analysis were performed to assess the myocardial damage and molecular mechanisms.

**Results:**

Kinase inhibition profiling analysis demonstrated that mitogen-activated protein kinases (MAPKs) mediated the signalling cascades of YQFM during HF therapy. Meanwhile, p38 and extracellular signal-regulated kinases (ERK1/2) were inhibited after YQFM treatment in H9c2 cells. In rats, the control group had lower left ventricular ejection fraction (LVEF) at 37 ± 1.7% compared with the YQFM group at 54 ± 1.1% (*p* < 0.0001). Cardiac fibrosis levels in control group rats were significantly higher than YQFM group (30.5 ± 3.0 vs. 14.1 ± 1.0, *p* < 0.0001).

**Conclusions:**

Our collective *in vitro* and *in vivo* experiments demonstrated that YQFM improves left ventricular (LV) function and inhibits fibrosis in HFrEF rats by inhibiting MAPK signalling pathways.

## Introduction

Heart failure (HF) is characterized by an impaired ability of the heart to pump blood to keep up with the circulatory demands of the body. Patients with HF encounter a classic collection of symptoms that reduce their quality of life, including dyspnoea, poor exercise tolerance and peripheral oedema (Ziaeian and Fonarow 2016). Management of high-risk factors (e.g., obesity, diabetes and poor diet) is the primary prevention strategy for cardiovascular diseases while angiotensin-converting enzyme inhibitors, β blockers, angiotensin-receptor blockers, mineralocorticoid receptor antagonists and diuretic treatment are the primary therapeutic drugs, possibly reducing HF-associated morbidity and mortality in several countries (Owens and Jessup 2011). Despite these advances, a declining trend has been observed in high-income countries or regions with regard to cardiovascular health (Zhao et al. 2015).

Once initiated, HF becomes a life-threatening syndrome, resulting in considerable burden on patients and healthcare systems. HF accounts for about 1–4% of all hospital admissions in economically developed countries (Shabu and Jayasekara 2020). Globally, 17–45% of patients die within the first year of initial admission (Ponikowski et al. 2014). The mean length of hospital stay for patients with HF is approximately 5–10 days (Callender et al. 2014). In low- and middle-income countries, patients with HF require prolonged hospital stays. Repeated hospital stays are typically required by patients with HF, which has been estimated to a total cost of $30.7 billion in 2012 in America alone (Heidenreich et al. 2013). Of this economic burden, more than two-third was attributable to direct medical costs (Heidenreich et al. 2013). Moreover, the costs associated with treating concomitant organ dysfunction are also anticipated to constitute a large portion of the total healthcare costs (Cook et al. 2014). Thus, there is an urgent need as well as a growing market for affordable therapies for HF.

Injection of YiQiFuMai (YQFM) powder is a modern Chinese medical preparation of natural products based on the historically proven Traditional Chinese Medicine. In China, it has been considered as a supplementary therapeutic strategy for treatment of HF with comparable ease of production and resupply, and patient compliance. YQFM, a concoction of concentrated plant extracts, consists of water-soluble compounds extracted from herbs, including the root of *Panax ginseng* C. A. Mey. (Araliaceae), the root of *Ophiopogon japonicus* (L. f) Ker-Gawl. (Liliaceae) and the fruit of *Schisandra chinensis* (Turcz.) Baill (Schisandraceae) (Yuan et al. 2011). YQFM's bioactive components have been identified, with ginsenosides and lignans being the most common pharmacologically active molecules (Zhou et al. 2011).

Currently available evidence reveals that YQFM has significant clinical efficacy for improving HF by enhancement of myocardial contractility (Nie et al. 2020), anti-inflammatory (Xing et al. 2013), anti-hypoxic (Feng et al. 2016) and apoptosis reduction (Li et al. 2016). Several studies have found associations between kinases (e.g., NF-κB, AMPKa and CaMKII) and the biological effects of YQFM. In an effort to uncover the pharmacological mechanism participating of YQFM against HF, we conducted its broadest kinome evaluation till date. The main aims of our study were: (1) to determine the kinase targets of YQFM and (2) perform *in vivo* and *in vitro* analyses to determine whether the therapeutic effect of YQFM on heart structure and function is mediated by these pivotal kinases signalling pathways.

## Materials and methods

### Drugs and reagents

YQFM for this study was provided by Tasly Pharmaceutical Co. Ltd. (Tianjin, China) (batch number of 20190314). Each 0.65 g of YQFM contains the ethanol extract (78 °C) of 0.5 g *Panax ginseng* dry root, 1.5 g *Ophiopogon japonicus* dry root and 0.75 g *Schisandra chinensis* dry fruit. The following antibodies and reagents were used: p38 mitogen-activated protein kinase (MAPK) (14064-1-AP, Proteintech, Shanghai, China), Phospho-p38 MAPK (P-p38) (4511S, Cell Signaling Technology, Boston, MA), p44/42 MAPK (Erk1/2) (4695S, Cell Signaling Technology, Boston, MA), Phospho-p44/42 MAPK (P-Erk1/2) (4370S, Cell Signaling Technology, Boston, MA), JNK Antibody (66210-1-Ig, Proteintech, Shanghai, China), Phospho-SAPK/JNK (4668S, Cell Signaling Technology, Boston, MA), MEK5 (15758-1-AP, Proteintech, Shanghai, China), MEK3 + MEK6 (ab200831, Abcam, Cambridge, MA), MEK1 + MEK2 (178876, Abcam, Cambridge, MA), HRP-labelled Goat Anti-mouse IgG (A0192, Beyotime Biotechnology, Shanghai, China), HRP-labelled Goat Anti-Rabbit IgG (A0208, Beyotime Biotechnology, Shanghai, China), FITC-labelled Goat Anti-Mouse IgG (A0428, Beyotime Biotechnology, Shanghai, China), Cy3-labeled Goat Anti-Rabbit IgG (A0516, Beyotime Biotechnology, Shanghai, China), RIPA Lysis Buffer (20188, Millipore, Billerica, MA), protease inhibitor cocktails (P8340, Sigma-Aldrich, St. Louis, MO), phosphatase inhibitor cocktail 2 and 3 (P5726, and P0044, Sigma-Aldrich, St. Louis, MO) and p38 inhibitor SB203580 (S1863, Beyotime Biotechnology, Shanghai, China).

### UPLC–MS analysis

The quality of YQFM was confirmed by high-performance liquid chromatography connected with a mass spectrometer (HPLC–MS) analysis. The ultra performance liquid chromatography (UPLC) analysis was carried out on Waters ACQUITYTM UPLC I-Class system (Waters, Milford, MA). The chromatographic separation was carried out on a Waters Symmetry C18 column (4.6 mm × 259 mm, 5 μm). The column temperature was set at 30 °C. The mobile phase flow rate was set at 1.0 mL/min, and the injection volume was 10 μL for each run. The mobile phase and line gradient program were handled as reported previously (Zhang et al. 2019b). The MS analysis was carried out by a Waters SYNAPT G2-SI MS system (Waters, Milford, MA). The analysis parameters were set as follows: the capillary voltage was 3.0 kV and the cone voltage was set at 40 V; the source temperature was 110 °C, and the desolvation gas temperature was 350 °C; the cone gas flow was 50 L/h, and the desolvation gas flow was 800 L/h. The MS data were acquired by Waters MassLynx V4.1 software and processed by UNIFI™ 1.8 software.

### Kinome-wide binding affinity screening and functional enrichment analyses

Kinome-wide annotation of YQFM selectivity was determined using the scanELECT services provided by DiscoverX. The YQFM was tested at a concentration of 2 mg/mL against a selected kinase panel containing a set of 408 kinases. The binding affinities of YQFM projected onto the human kinome tree were generated using the TREEspot software tool. Data are presented as the percentage of control activity remaining. Kinases with inhibition rates ≥40 were collected from kinome-wide binding affinity screening data. Functional enrichment analyses were performed to understand the advanced functions of these kinases in the biological systems. Meaningful interpretation of the biological information on the selected kinases was performed using the DAVID database.

### Cell culture

Rat H9c2 cardiomyocyte cell line was purchased from the Shanghai Institute of Cell Biology (Shanghai, China). The H9c2 cells were maintained in Dulbecco's modified Eagle medium (DMEM) supplemented with 10% foetal bovine serum (FBS), 100 U/mL penicillin and 100 μg/mL streptomycin at 37 °C in a humidified atmosphere of 5% CO_2_ and 95% air. The culture medium was replaced every two days, and cells were subjected to increasing concentrations of YQFM and positive inhibitor (p38 inhibitor, SB203580) for 24 h at 80–90% confluence.

### Western blotting

H9c2 cells were incubated with YQFM (0, 0.5, 0.75, 1.0, 1.5, 2.0, 3.0 and 4.0 mg/mL) for 24 h. H9c2 cardiomyocytes were rinsed twice with PBS buffer and lysed with a lysis buffer for Western blotting. Western blotting analysis of LV tissue was performed as described previously (Nie and Liu 2017). Briefly, 10–20 μg protein was transferred onto polyvinylidene difluoride (PVDF) membranes. The PVDF membrane was incubated with primary antibodies at 4 °C overnight, followed by incubation with secondary antibodies. The protein–antibody complexes were washed three times with TBST buffer, followed by visualization using a Tanon 5200 detection system.

### Immunofluorescence staining and microscopy

Indirect immunofluorescence was performed as described previously (Nie et al. 2016). Briefly, H9c2 cells were seeded in 24-wells. H9c2 with or without 24 h YQFM treatment (2.0 mg/mL) were rinsed twice with PBS buffer and fixed in paraformaldehyde at room temperature, after permeabilized by Triton X-100, saturated with BSA, and incubated overnight with the p38, P-p38, ERK1/2 and P-ERK1/2 antibodies and appropriate fluorescent labelling of secondary antibodies. Nuclei were stained with 4′,6-diamidino-2-phenylindole (DAPI). Images were acquired using a Nikon A1R laser scanning confocal microscope.

### Animals

Male Sprague-Dawley rats weighing 220–250 g were obtained from Beijing Vital River Laboratory Animal Technology Co., Ltd (Beijing, China). The animals were housed individually and placed at a constant temperature of 23 ± 1 °C in a 12 h light/dark cycle room. Commercial standard solid rodent chow and water were freely available throughout the experiments. All the research protocols for the present study adhered to the guidelines of the National Research Council Guide for the Care and Use of Laboratory Animals. This study was approved by the Tasly Holding Group’s Animal Care and Use Committee (TSLZJ2020009).

### Animal treatment and grouping

A model of HF with reduced ejection fraction (HFrEF) was produced secondary to permanent ligation of left anterior descending (LAD). Successful ligation of the LAD was confirmed by the occurrence of ST-segment elevation on electrocardiography. Sham operated rats were subjected to the same process without ligation of the LAD. The successfully ligated rats were maintained for a period of 6 weeks and live rats with ejection fraction (EF) ≤40 were randomly separated into the control group and YQFM group. YQFM powder was dissolved in 0.9% sodium chloride. (1) Sham group: sham operated rats (*n* = 10) were administrated 0.9% sodium chloride for 14 days via tail intravenous injection at the dose of 2 mL; (2) control group: model operated rats (*n* = 20) were administrated 0.9% sodium chloride for 14 days via tail intravenous injection at the dose of 2 mL; and (3) YQFM group: model operated rats (*n* = 20) were administrated 0.46 g/kg/d YQFM for 14 days via tail intravenous injection.

### Echocardiography measurements

After 14 days of treatment, transthoracic echocardiography was performed under anaesthesia (2% isoflurane, inhalation anaesthetics) using a Sonoscape S9 Portable Color Doppler Diagnostic Scanner System with a 15-MHz probe (SonoScape Medical Corp., Shenzhen, China). The following parameters were measured: left ventricular internal diameter at end-diastole (LVIDd), left ventricular internal diameter at end-systole (LVIDs), left ventricular volume at end-diastole (LVVd) and left ventricular volume at end-systole (LVVs). Person administering the transthoracic echocardiography does not know whether it is the placebo or the YQFM treatment. All data were measured thrice. The mean value was used. Fractional shortening (FS) was calculated as follows: FS=(LVIDd – LVIDs)/LVIDd × 100. The EF was calculated as follows: EF=(LVVd – LVVs)/LVVd × 100.

### Histological analysis

After 14 days of treatment, the rats were euthanized; the hearts were fixed in 4% paraformaldehyde. Haematoxylin and eosin (H&E) and Masson’s trichrome staining were performed. Myocardial fibrosis was assessed in 10 fields per sample by two independent researchers to obtain a mean value. Immunohistochemistry analysis was performed using primary antibodies against P-p38 MAPK and P-ERK1/2 MAPK at 4 °C for 24 h. After washing, the sections were incubated with secondary antibody at 37 °C for 1 h. The sections were observed under a light microscope (Motic Digital Pathology, San Francisco, CA).

### Statistics analyses

Statistical analyses were performed using GraphPad Prism version 8.04 for Windows software (La Jolla, CA). Data are presented as the mean ± SD. Statistical analysis was performed using an unpaired, two-tailed Student’s *t*-test between two groups. Statistical significance was set at *p* < 0.05.

## Results

### Chemical characterization in 20190314 batches of YQFM

**Figure 1.**
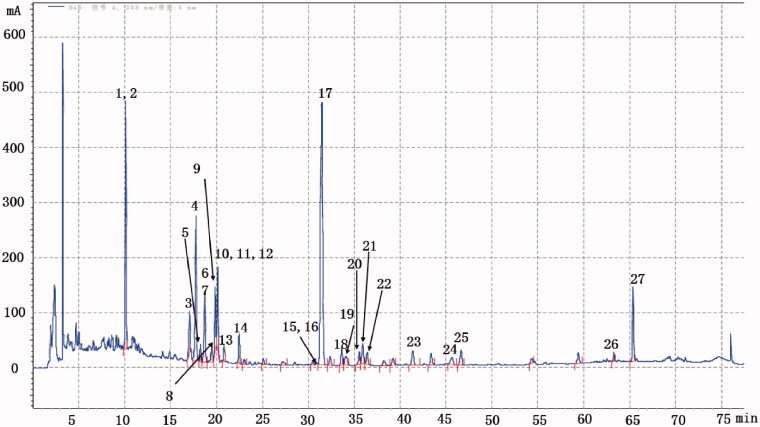
Identification of main components in 20190314 batches of YQFM. (1) Ginsenoside Re; (2) ginsenoside Rg1; (3) ginsenoside Rf; (4) ginsenoside Rb1; (5) notoginsenoside R2; (6) ginsenoside F3; (7) ginsenoside Rc; (8) ginsenoside Rg2; (9) ginsenoside Rb2; (10) ginsenoside Rg2; (11) ginsenoside Ro; (12) ginsenoside Rb3; (13) 20(S)-ginsenoside Rh1; (14) (20S)-ginsenoside Rd; (15) ginsenoside Rg6; (16) 20(s)-ginsenoside F2; (17) schizandrin A; (18) ginsenoside Rh4; (19) taiwanschirin D; (20) angeloylgomisin Q; (21) ginsenoside Rg3; (22) schizandrol B; (23) tigloylgomisin H; (24) ginsenoside Rk1; (25) ginsenoside Rg5; (26) alpha-linolenic acid; (27) conjugated linoleic acid.

Representative chromatograms of 20190314 batches of the YQFM using the UPLC method are shown in Figure 1. In this study, 27 compounds were identified and characterized based on their retention times and MS spectra. The UPLC profiles showed that the active components in YQFM were stable in 20190314 batches.

### Inactivation of MAPKs was the prominent signalling cascade induced by YQFM

**Figure 2.**
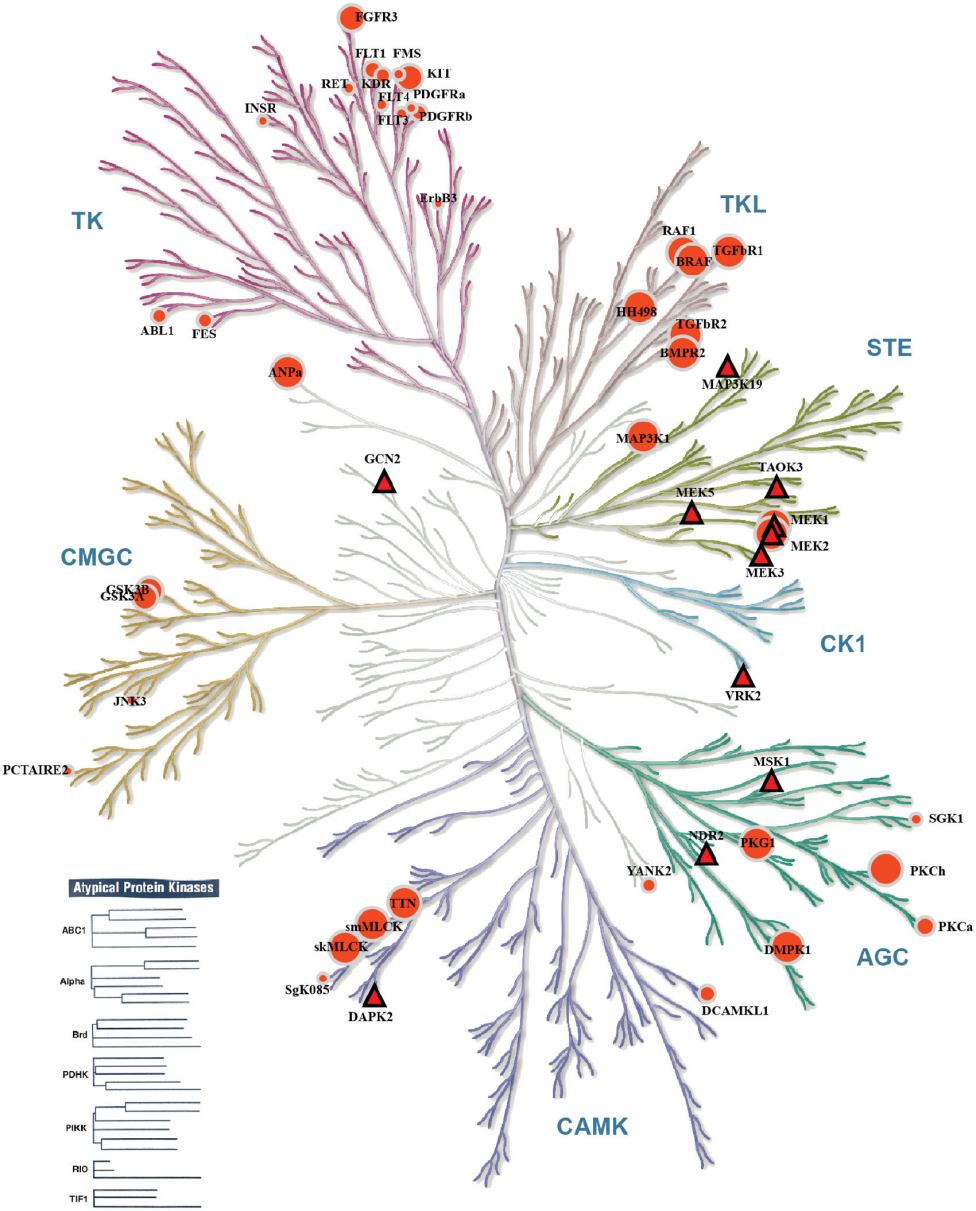
Kinases complying with inhibition rates ≥40 shown on the human kinome dendrogram. The top kinases inhibited by YQFM were determined using the KinaseProfiler of DiscoverX (red triangle). The 39 kinase-disease association therapeutic area of cardiovascular disease were developed by built-in data Sources of KinMap (OrangeRed circle). The figure was generated by using an online KinMap program (http://kinhub.org/kinmap/).

YQFM displayed no inhibitory activity against 26% of the human protein kinases. Twelve kinases were selected from the screening data, complying with inhibition rates ≥40 (Table 1). The complete data of kinase inhibition profiling assays against a series of 408 kinases performed through Eurofins kinase profiling are presented in Supporting Information (Table S1). As shown in Figure 2, MEK1 and MEK2 inhibitory activity of YQFM was associated with the kinase-disease association therapeutic area of cardiovascular disease in KinMap Data Sources. KEGG pathway enrichment analysis suggested that six of the kinases with inhibition rates ≥40 were enriched in the MAPK signalling pathway, while apoptotic process and regulation of autophagy involved DAPK2. TAOK3, MAP3K19, MSK1, MEK3, VRK2, MEK1, MEK5 and MEK2 were the top individual kinases that correlated most closely with MAPKs signalling cascades. In light of these facts, we hypothesized that YQFM has pharmacological effects against MAPKs signalling cascades during HF therapy.

**Table 1.** Kinases complying with study criteria of inhibition rates ≥40.

Kinase	Functional annotation	Inhibition rates (%)
MAPK signalling pathway (KEGG_PATHWAY)		
TAOK3	Positive regulation of stress-activated MAPK cascade	57
MSK1(RPS6KA5)	MAPK signalling pathway, adrenergic signalling in cardiomyocytes	54
MEK3 (MAP2K3)	Activation of MAPK activity	50
MEK1 (MAP2K1)	Activation of MAPK activity, heart development, positive regulation of ERK1 and ERK2 cascade	44
MEK5 (MAP2K5)	Activation of MAPK activity, heart development, negative regulation of NF-kappaB transcription factor activity	42
MEK2 (MAP2K2)	Activation of MAPK activity, positive regulation of ERK1 and ERK2 cascade	40
MAP3K19 (YSK4)	Signal transduction by protein phosphorylation	57
VRK2	Cellular response to oxidative stress, regulation of MAPK cascade	48
Cell death		
DAPK2	Apoptotic process, regulation of autophagy	54
Protein processing		
EIF2AK4 (GCN2)	Negative regulation of translational initiation in response to stress	40
Inositol phosphate metabolism		
PIP4K2B	Inositol phosphate metabolism, phosphatidylinositol signalling system	41
Cellular component organization		
STK38L (NDR1)	Intracellular signal transduction, regulation of cellular component organization	48

### YQFM induces p38 and ERK1/2 inhibition in H9c2 cardiomyocytes

**Figure 3.**
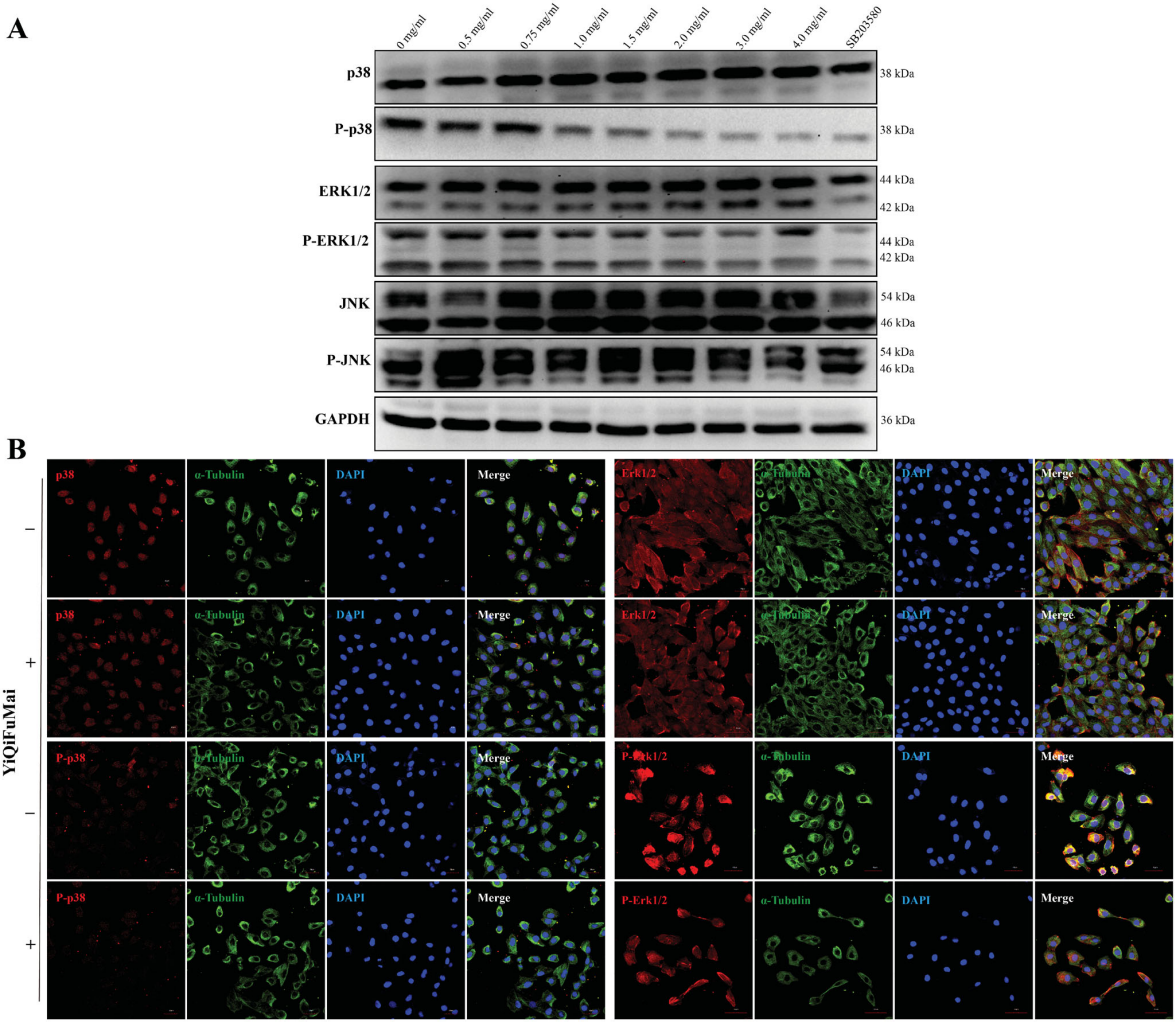
Effects of YQFM on MAPKs signalling cascades in H9c2 cardiomyocytes. (A) Representative Western blotting images of activation status of three major branches of MAPKs in H9c2 cardiomyocytes treatment with increasing concentrations of YQFM, and p38 inhibitors SB203580. (B) Immunofluorescence staining demonstrating the effects of 2 mg/mL YQFM on P-p38 MAPK, P-ERK1/2, p38 and ERK1/2 expression in H9c2 cardiomyocytes. Scale bars = 30–50 μm.

Next, we wanted to examine how this YQFM induced MAPKs inhibition translated to cardiomyocytes. Western blotting analysis was carried out to measure the activation status of p38, ERK1/2 and JNK in H9c2 cardiomyocytes treated with YQFM. The activation status of p38 and ERK1/2, as indicated by the phosphorylated versus the total protein levels, exhibited dose-dependent reductions after YQFM treatment. In addition, neither JNK activation nor total protein levels changed significantly during YQFM treatment. MAPKs were differentially inhibited by YQFM in H9c2 cardiomyocytes, and inactivation of p38 MAPK in cardiomyocytes was consistent with the known action of the p38 inhibitor SB203580 (Figure 3(A)). We also analysed the p38 and ERK1/2 activation states of YQFM treatment by conducting an indirect immunofluorescence analysis. YQFM-treated cells showed diminished p38 and ERK1/2 activation levels compared with untreated controls, which is consistent with the results of Western blotting analysis (Figure 3(B)).

### YQFM improved the LV function in LAD permanent ligation-induced HF rats

**Figure 4.**
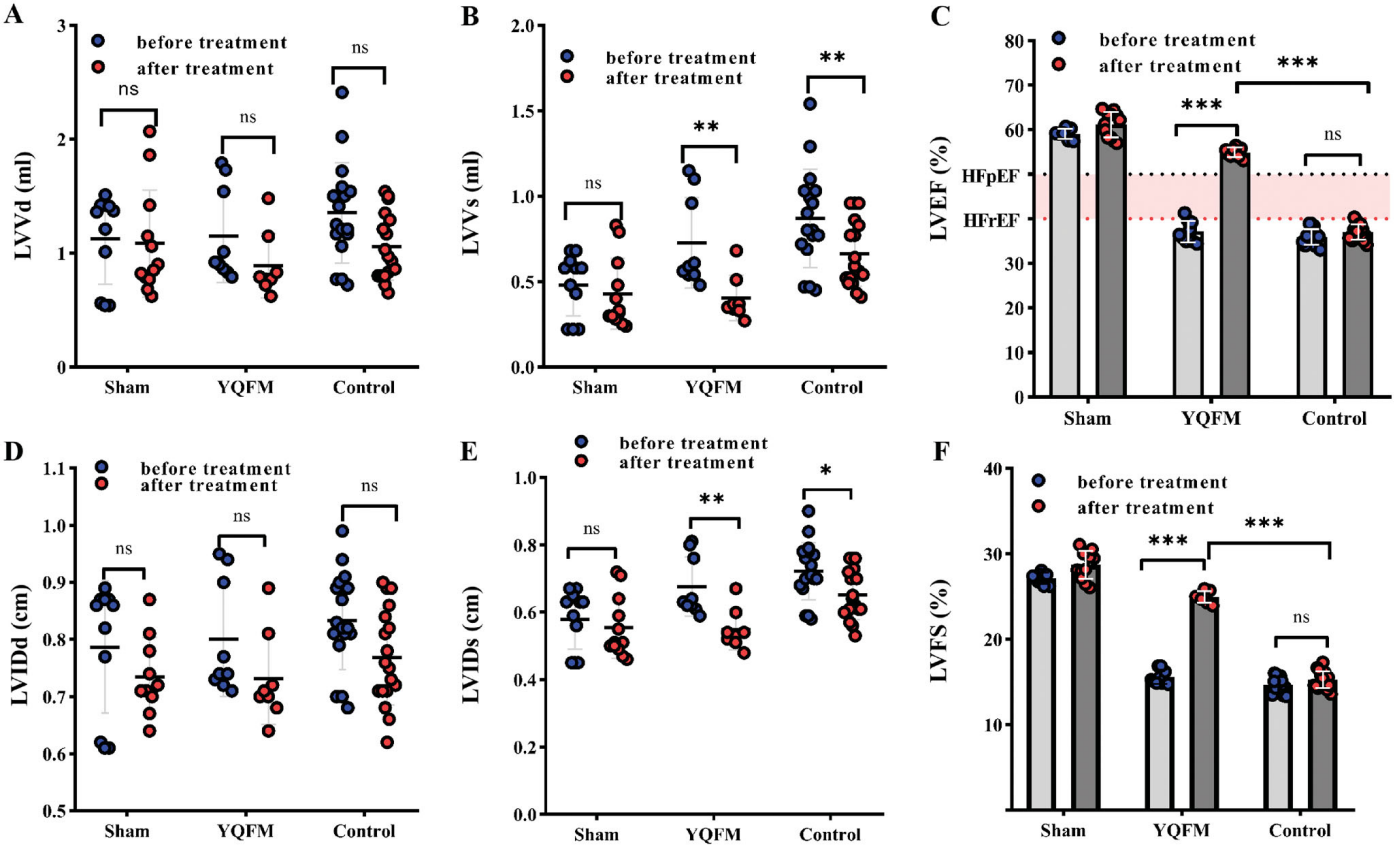
Echocardiogram analysis showing YQFM improvement of LV function in HFrEF rats. Serial echocardiographic measurements of (A) Left ventricular volume at end-diastole, (B) left ventricular volume at end-systolic, (D) left ventricular internal diameter at end-diastole and (E) left ventricular internal diameter at end-systole in the sham, control and YQFM group rat hearts before and after treatment. LV echocardiogram parameters included (C) ejection fraction (EF) and (F) fractional shortening (FS) were calculated as indicated (sham *n* ≥ 8, model *n* ≥ 17, YQFM *n* ≥ 8). Data are indicated as mean ± SD, ***p < 0.0001, **p < 0.01, *p < 0.05 and ^ns^*p* > 0.05.

To assess whether YQFM modulates cardiac function, model operated rats with HFrEF were treated with the YQFM (0.46 g/kg/d) for 14 days. After treatment, LVVd (Figure 4(A)) and LVIDd (Figure 4(D)) were not significantly altered in HFrEF rats treated with YQFM. Left ventricular ejection fraction (LVEF) and LVFS were not significantly altered in HFrEF rats treated with placebo; however, YQFM caused a significant increase in EF (Figure 4(C)) and FS (Figure 4(F)), indicating improvement of LV function. The improvement of LV function in response to YQFM was accompanied by a decrease in LVVs (Figure 4(B)) and LVIDs (Figure 4(E)).

### YQFM induces MAPKs inhibition in LAD permanent ligation-induced HFrEF rats

**Figure 5.**
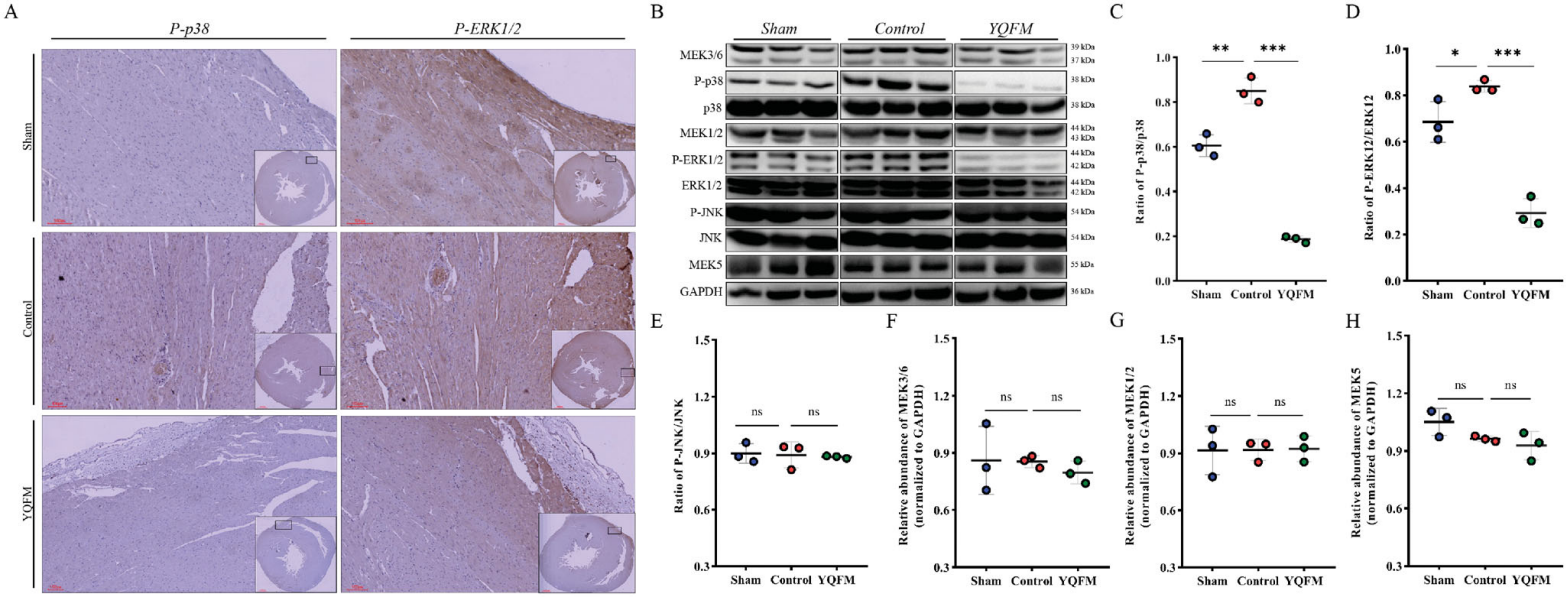
Effects of YQFM on MAPKs signalling cascades in rats with HFrEF induced by LAD permanent ligation. (A) Immunofluorescence staining demonstrating P-p38 and P-ERK1/2 expression and localization in sham, control and YQFM group hearts. Western blotting images (B) and quantitative densitometry analysis of P-p38/p38 (C), P-ERK1/2/ERK1/2 (D), P-JNK/JNK (E), MEK3/6 normalized to GAPDH (F), MEK1/2 normalized to GAPDH (G) and MEK5 normalized to GAPDH (H) from LV tissue homogenates in HFrEF rats; *n* = 3 per group. Data are indicated as mean ± SD, ***p < 0.0001, **p < 0.01, *p < 0.05 and ^ns^*p* > 0.05.

To determine whether the improvement of cardiac function efficacy in response to YQFM in HFrEF rats could result from the direct impact of the inhibition on p38 and ERK1/2, we performed P-p38 MAPK and p-ERK1/2 immunohistochemical staining of hearts obtained from the placebo and YQFM-treated HFrEF rats. Immunohistochemical staining revealed that P-p38 MAPK and P-ERK1/2 expression was increased in the nucleus and cytoplasm of HFrEF rats compared to sham operated animals. YQFM treatment in comparison with placebo treatment caused a significant reduction in p38 and ERK1/2 activation (Figure 5(A)). Moreover, Western blotting analysis of LV tissue demonstrated that YQFM significantly downregulated the activation status of p38 and ERK1/2 compared with placebo (Figure 5(B–D)). However, the activation of JNK and total protein levels of MAPKs such as MEK5, MEK1, MEK2, MEK3 and MEK6 were not significantly altered in HFrEF rats treated with YQFM compared with placebo (Figure 5(E–H)).

### YQFM improved the cardiac fibrosis in LAD permanent ligation-induced HFrEF rats

**Figure 6.**
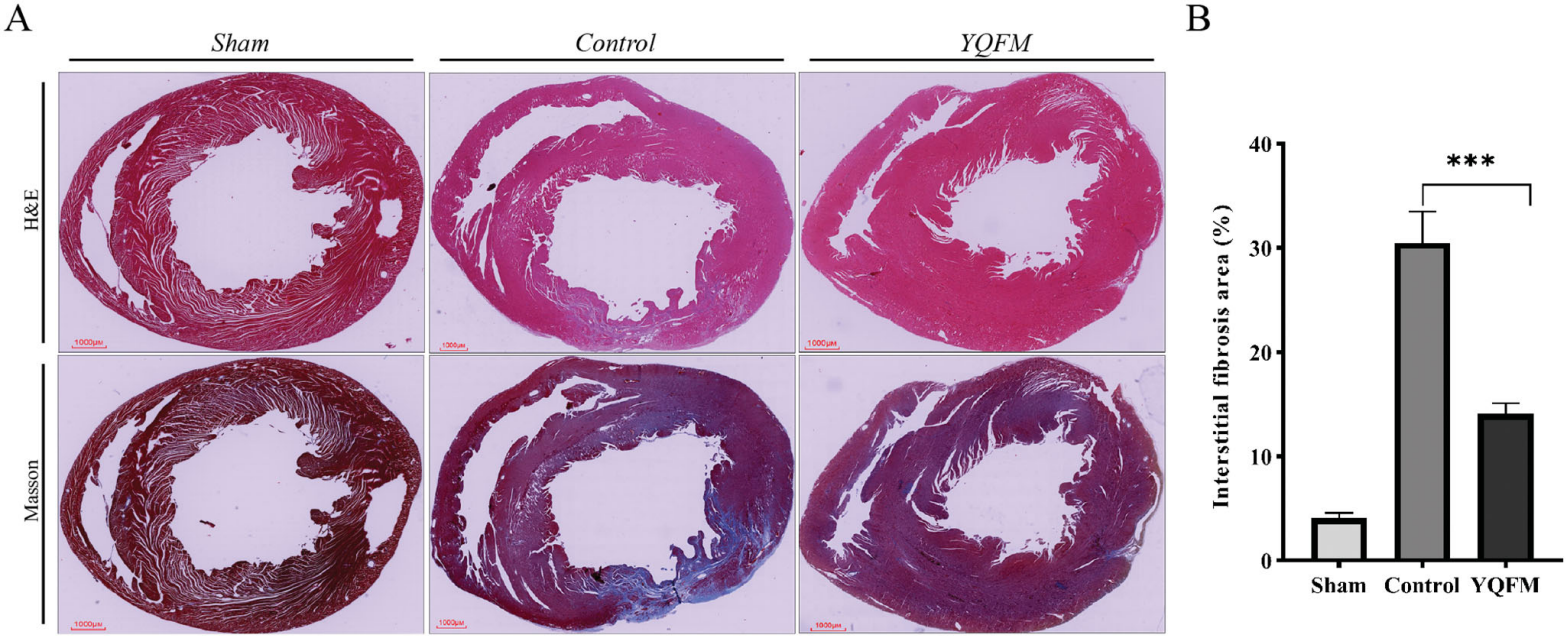
Effects of YQFM on cardiac fibrosis in rats with HFrEF. (A) Representative H&E-stained and Masson-stained whole-heart images of sham, control and YQFM group hearts at day 56 after surgery (scale bars = 1000 μm). (B) Fibrosis area (%) of sham, control and YQFM group hearts at day 56 after surgery (sham *n* = 8, model *n* = 12, YQFM *n* = 8; data are indicated as mean ± SD).

Next, we measured the fibrosis status of HFrEF rats, as described in ‘Materials and methods’ section. We observed orderly myocardial cells with normal myocardial interstitium in sham group. Compared with the sham group, the cardiomyocytes in the control group were disordered, and the intercellular spaces were wider. Masson's staining revealed deposition of collagen fibres (blue staining) in the myocardial interstitium (Figure 6(A)). Quantitatively, the percentage area of fibrosis in the model group hearts (30.5 ± 3.0%) was significantly greater than that in the YQFM group hearts (14.1 ± 1.0%) (Figure 6(B)). The histological benefits in response to YQFM were accompanied by a decrease in the percentage of total myocardial tissue occupied by collagen fibres and improvements in cardiomyocyte reduction.

## Discussion

In this report, we attempted to decipher the pharmacological pathways of YQFM acting on HF using a kinome-wide binding affinity screening strategy. We found that p38 and ERK1/2 were prominently and dynamically inhibited in a dose-dependent manner after YQFM treatment and were associated with improved LV function and lower levels of cardiac fibrosis in YQFM treated versus placebo treated LAD permanent ligation-induced HFrEF rat hearts.

HF impacting cardiovascular health is multi-factorial and has been shown to divide into subgroups based on LVEF being either reduced or preserved (Figtree et al. 2021). Myocardial changes in HF can include deterioration in the force of contraction, adaptive hypertrophic growth and alterations in fibrosis (Arabacilar and Marber 2015). The emerging role of MAPKs in human in response to myocardial infarction-induced HF has been demonstrated in several studies (Kumphune et al. 2015). Kinases that are involved in the MAPK pathway have been reported to be activated in patients with advanced HF (Haq et al. 2001; Woulfe et al. 2020). In a mouse model of left ventricular (LV) pressure overload, time-dependent MAPK activation has also been reported (Esposito et al. 2001; Ai et al. 2019). Moreover, cardiac-specific activation of p38 MAPK markedly attenuated cardiac contractility (Liao et al. 2002; Yokota et al. 2020). Cardiac fibroblast-specific p38α MAPK contributes to cardiac hypertrophy and TGF-β signalling-mediated cardiac fibrosis (Molkentin et al. 2017).

Natural products from the root of *Panax ginseng*, the root of *Ophiopogon japonicus*, and the fruit of *Schisandra chinensis* have been used to treat cardiovascular diseases for thousands of years in China. During that time, medicinal herbs were empirically applied to influence overall outcomes, without knowledge of their specific biological effects or pharmacological activities. In the experiments presented here, YQFM-specific inactivation of p38 and ERK1/2 during HF development is one of the key findings. LV function was ameliorated in HFrEF hearts, which likely contributed to a decrease in LVVs and LVIDs concomitant with reduced fibrosis. In line with a recently published report (Pang et al. 2017), we confirmed that YQFM-induced MAPKs inhibition is associated with an anti-HF effect. In addition, our data indicate the inhibition of MEK5 MAPK in a human kinase inhibitor binding platform treated with YQFM. This MAPK-specific inhibition by YQFM raises attention to its therapeutic role in MAPK-specific pathogenesis in HF.

Currently, cardiovascular disease drug discovery strategies tend to identify single compound-based leads. However, substantial gaps remain in our pharmacotherapy armament for HF therapy (Figtree et al. 2021). The relative failure of drug solution may partly be attributed to the broad spectrum of phenotypes and non-cardiac comorbidities (Triposkiadis et al. 2016). Plant-based natural products in YQFM comprise of identified bioactive molecules against cardiovascular diseases. Ginsenosides, the major active components of *Panax ginseng*, have been shown to protect the heart from ischaemic and ischemia–reperfusion injury (Karmazyn et al. 2011). Polysaccharides from *Ophiopogon japonicus* have been shown to alleviate heart injury in diabetic rats (Zhang et al. 2016). Another important ingredient in YQFM, *Schisandra chinensis* has been reported to protect against lipopolysaccharide-induced H9c2 cell injury through major bioactive schizandrin (Zhang et al. 2019a). Considering that HF results from several different aetiological pathways, the multi-kinase target of YQFM treatment may be more effective in treating HF than single compound-based drugs. Further high-throughput methods for evaluation, isolation and purification of active compounds are needed to be performed, as this will allow scientists to determine the molecular basis, safety and pharmacokinetics of the therapeutic effect of YQFM.

## Conclusions

In this study, for the first time, we demonstrated that YQFM inhibits ERK1/2 and p38 activation using a kinome-wide binding affinity screening strategy. Our collective *in vitro* and *in vivo* experiments confirmed that YQFM improved LV function and inhibited cardiac fibrosis partly by direct impact of the phosphorylation of MAPK signalling pathways. To our knowledge, this is the first evidence that YQFM-kinase phosphorylation is an important pharmacological regulator of HF. We developed a hypothesis about the molecular mechanisms of YQFM on HFrEF, but the precise active components and/or component concoction are still unknown. More rigorous studies are needed to confirm this in the future.

## Author contributions

Dekun Li supervised the study. Yongwei Nie and Yanxin Zhang designed and performed the experiments. Zhi Li and Meixu Wan participated in performing the analysed data. Yongwei Nie wrote the paper draft and finalized the manuscript. All authors agree to be accountable for all aspects of work ensuring integrity and accuracy.

## Supplementary Material

Supplemental Material

## Data Availability

The data underlying this article will be shared on reasonable request to the corresponding author.
